# Spinal Tuberculosis Mimicking Metastatic Lung Cancer: A Case of Misdiagnosis

**DOI:** 10.7759/cureus.91815

**Published:** 2025-09-08

**Authors:** Sahak Mkrtchyan

**Affiliations:** 1 Department of Tuberculosis, Yerevan State Medical University after Mkhitar Heratsi, Yerevan, ARM

**Keywords:** biopsy diagnosis, drug-resistant tb, extrapulmonary tuberculosis (eptb), infection mimicking malignancy, mdr tuberculosis, metastatic lung cancer, pott’s disease-tuberculous spondylitis, spinal tuberculosis, thoracic spine lesions, tumor misdiagnosis

## Abstract

Misdiagnosis of spinal tuberculosis (TB), otherwise known as Pott's disease, can lead to inappropriate treatments and prolonged morbidity. Spinal TB may mimic malignant lesions on imaging, and, in addition, its chronic course, weight loss, and constitutional symptoms can further contribute to misdiagnosis, occasionally resulting in inappropriate chemotherapy or radiotherapy before the correct diagnosis is established. A 44-year-old woman presented with progressive thoracic spine pain, lower limb radiation, weight loss, and intermittent low-grade fever. She was initially misdiagnosed with a lung neoplasm with spinal metastases based on imaging and received chemotherapy and radiotherapy without improvement. Definitive diagnosis was made via thoracoscopic biopsy of lung and spinal lesions, revealing rifampicin- and isoniazid-resistant TB. Imaging confirmed destructive thoracic vertebral lesions with prevertebral and epidural extension, consistent with spinal TB with large cold abscesses. The patient was treated with a tailored multidrug-resistant TB regimen, including bedaquiline, linezolid, levofloxacin, clofazimine, and cycloserine, alongside supportive therapy. She tolerated the treatment well without significant adverse effects. This case highlights the diagnostic challenge of spinal TB mimicking malignancy and underscores the importance of biopsy for accurate diagnosis and the timely initiation of appropriate therapy.

## Introduction

Tuberculosis (TB) continues to pose a significant global health burden despite sustained control efforts and international targets for its elimination [1], and it remains a notable public health concern in the South Caucasus region. Nonetheless, efforts to eradicate TB have been hindered by socioeconomic disparities, shifting public health priorities, and unforeseen challenges such as the COVID-19 pandemic. Among extrapulmonary forms, spinal TB is the most common, accounting for nearly half of all musculoskeletal TB cases [2]. Spinal TB remains a significant cause of disability and suboptimal outcomes, often stemming from delayed diagnosis or inadequate treatment [3].

Diagnosis of spinal TB is often delayed due to its nonspecific clinical presentation and radiological resemblance to other pathologies [4]. In particular, vertebral lesions may closely mimic metastatic cancer on imaging, which can lead to inappropriate treatments such as chemotherapy or radiotherapy before TB is considered [5]. The presence of drug-resistant TB further complicates management.

This report describes a rare presentation of rifampicin- and isoniazid-resistant spinal TB initially mistaken for metastatic lung cancer, resulting in the delayed initiation of appropriate therapy. The case illustrates the potential for spinal TB to mimic malignancy, the risks of misdiagnosis, and the importance of timely histopathological and microbiological assessment to ensure accurate diagnosis and targeted treatment.

## Case presentation

A 44-year-old woman was admitted to the Extrapulmonary Department of the National Pulmonology Center after being referred from a regional medical center, where she had been evaluated for persistent respiratory and spinal symptoms. She had a three-year history of progressive thoracic spine pain radiating to both lower limbs, physical activity limitation, general weakness, weight loss, and intermittent low-grade fever. Over this period, she had been seen in multiple medical centers and, approximately two years prior, was incorrectly diagnosed with a neoplasm of the right upper lung lobe with metastases to the thoracic spine. Based on this presumed malignant process, she underwent chemotherapy and radiotherapy, but her condition continued to deteriorate.

Based on the patient's lack of clinical improvement and progressive deterioration despite oncologic treatment, a thoracoscopic biopsy of lung tissue and the adjacent spinal lesion was performed. Histopathological and microbiological examination of the obtained biomaterial revealed a tuberculous lesion with confirmed resistance to rifampicin and isoniazid. Following this diagnosis, the patient was referred to the National Pulmonology Center for specialized management.

Upon admission, the patient was in a moderate state of general health. She was conscious and oriented, with stable vital signs: a blood pressure of 120/80 mmHg (measured manually), a pulse rate of 67 beats per minute, and an oxygen saturation within normal limits. The physical examination revealed pale but clean skin without peripheral edema. The cardiovascular examination revealed clear and rhythmic heart sounds. Lung auscultation demonstrated vesicular breath sounds bilaterally, without any adventitious sounds. The abdomen was soft and non-tender on palpation, with normal bowel and bladder function.

Laboratory investigations on admission revealed that the complete blood count parameters were within normal limits, except for a mildly elevated erythrocyte sedimentation rate. Blood biochemistry results were generally unremarkable, with all tested values falling within normal ranges. Human immunodeficiency virus and viral serology tests were negative, indicating no underlying immunodeficiency. Additional laboratory data are presented in Table 1.

**Table 1:** Laboratory findings on admission.

Test	Result	Reference range	Interpretation
Complete blood count			
Hemoglobin	126 g/L	120-160 g/L	Normal
Red blood cells	4.8×10¹²/L	4.2-5.4×10¹²/L	Normal
Platelets	281×10⁹/L	150-400×10⁹/L	Normal
White blood cells	7.66×10⁹/L	4.0-11.0×10⁹/L	Normal
Neutrophils	52.1%	40-70%	Normal
Lymphocytes	37.2%	20-40%	Normal
Monocytes	8.1%	2-10%	Normal
Eosinophils	0.4%	1-6%	Low
Erythrocyte sedimentation rate	35 mm/hr	0-20 mm/hr	Mildly elevated
Blood biochemistry			
Total protein	60.5 g/L	60-80 g/L	Normal
Albumin	36.8 g/L	35-50 g/L	Normal
Creatinine	52 µmol/L	45-90 µmol/L	Normal
Uric acid	0.212 mmol/L	0.18-0.48 mmol/L	Normal
Alanine aminotransferase	21.8 U/L	7-56 U/L	Normal
Aspartate aminotransferase	16.2 U/L	5-40 U/L	Normal
Blood glucose	5.61 mmol/L	3.9-5.8 mmol/L	Normal
Total bilirubin	18.8 µmol/L	3.4-20.5 µmol/L	Normal

Ultrasound examination of the abdomen demonstrated normal findings, with no ascites and a minimal amount of fluid in the right sinus. Electrocardiograms performed on admission and during hospitalization showed a normal sinus rhythm without dynamic changes.

Chest X-ray revealed linear fibrotic changes in the right upper lung lobe, a limited air-containing area in the right apex, nodular shadows in the right middle and lower lung fields, thickening of the costal pleura, and fine fibrosis in the left lower lung field, with no sinus abnormalities.

Thoracic spine X-ray showed narrowing of the Th8-Th9 intervertebral space, destructive foci, and wedge-shaped deformities of the Th8 and Th9 vertebral bodies, accompanied by an adjacent paravertebral abscess, consistent with spinal TB.

**Figure 1:**
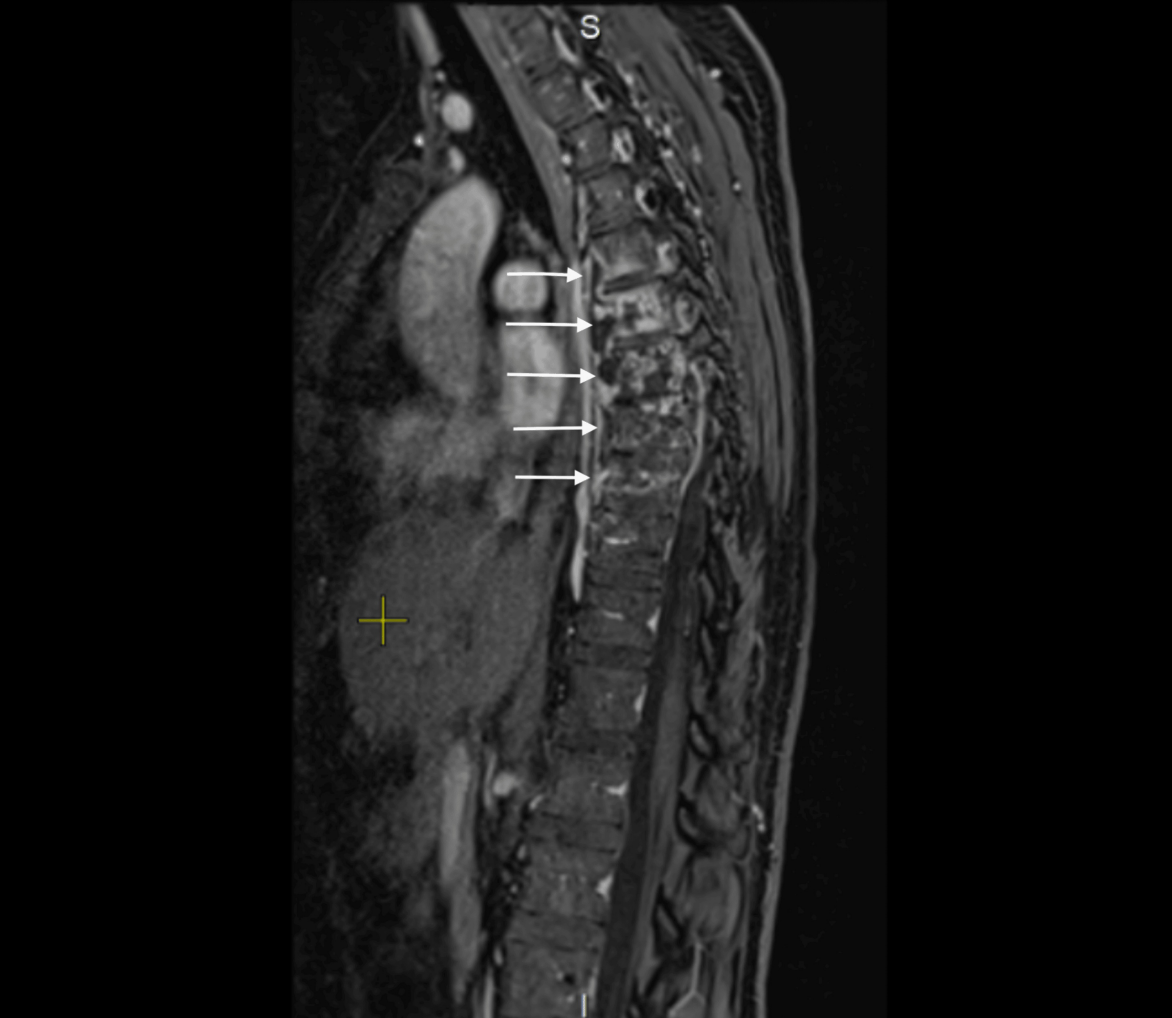
Sagittal MRI of the thoracic spine showing destructive vertebral lesions. White arrows indicate the affected vertebral bodies. MRI: magnetic resonance imaging

**Figure 2:**
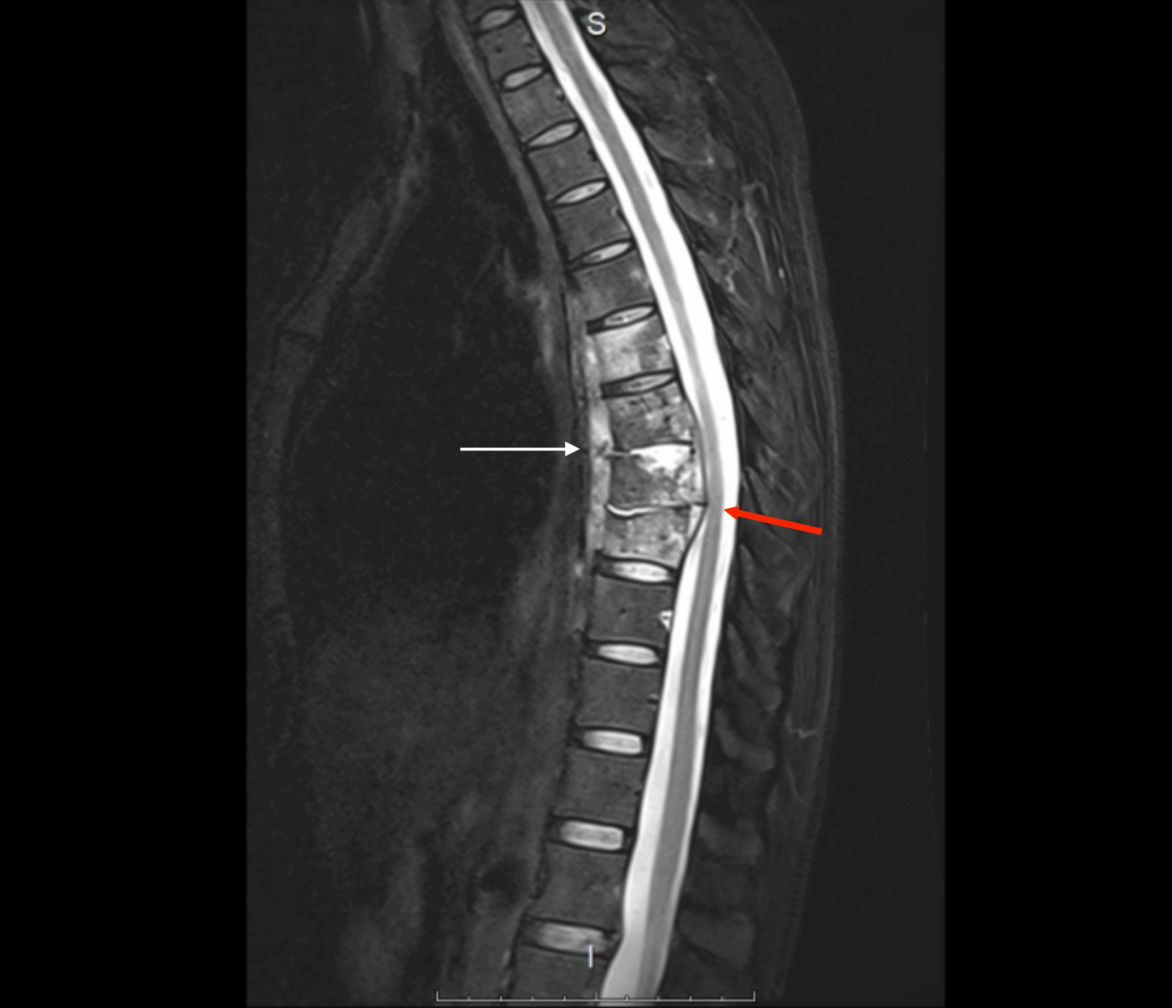
Sagittal MRI of the thoracic spine showing the prevertebral cold abscess. The white arrow indicates the abscess, and the red arrow indicates narrowing of the anterior subarachnoid space. MRI: magnetic resonance imaging

Magnetic resonance imaging (MRI) of the thoracic spine (Th5-Th10) revealed extensive vertebral body involvement, including a secondary fracture of Th8, with prevertebral extension forming large cold abscesses, showing peripheral contrast enhancement. At Th7-Th9, there was an epidural spread into the intraspinal space without transdural extension, causing marked narrowing of the anterior subarachnoid space and abutment of the spinal cord, without cord signal changes. Lesions extended into the intervertebral foramina with nerve root involvement and into the posterior basal segments of the right lung and adjacent ribs. The Th7-Th9 intervertebral discs were markedly reduced in height and hyperintense on T2, and the Th9 vertebral body demonstrated wedge-shaped deformation. MRI findings are shown in Figure 1 and Figure 2.

The patient was initiated on a directly observed treatment regimen tailored for multidrug-resistant TB, including bedaquiline, linezolid, levofloxacin, clofazimine, and cycloserine, with a planned duration of 18 months. She tolerated the treatment well throughout. Adjunctive therapy included vitamin B6 supplementation, gastroprotective agents, analgesics, and supportive infusion therapy. Strict bed rest was also maintained.

Following discharge, the patient was scheduled for a follow-up visit in two months to evaluate treatment response and clinical progress. Additionally, she was referred to an orthopedic surgeon for further management after completing the TB treatment.

## Discussion

In the present case, the patient's chronic back pain, weight loss, and imaging evidence of destructive vertebral lesions with paravertebral extension led to an initial diagnosis of metastatic lung cancer. This misdiagnosis resulted in exposure to chemotherapy and radiotherapy without clinical improvement. Similar instances have been documented where spinal TB was mistaken for malignancy due to overlapping imaging characteristics, including vertebral body destruction, paravertebral masses, and heterogeneous contrast enhancement [6,7]. The absence of classic pulmonary TB findings on chest radiography further complicated the clinical picture.

The delay in diagnosis highlights a critical issue: reliance on imaging alone is insufficient to distinguish spinal TB from other destructive vertebral pathologies. While MRI is the preferred modality for early detection due to its sensitivity in identifying bone marrow edema, abscess formation, and epidural involvement [8], these features are not specific. Histopathological and microbiological confirmation through biopsy remains the gold standard for diagnosis [9]. In our patient, thoracoscopic biopsy of lung and vertebral lesions was decisive, revealing *Mycobacterium tuberculosis* complex with rifampicin and isoniazid resistance. This underscores the necessity of obtaining tissue diagnosis before initiating oncologic or prolonged antimicrobial therapy in cases of atypical vertebral destruction.

Drug-resistant spinal TB presents unique therapeutic challenges. Rifampicin and isoniazid resistance, particularly when combined, significantly limit first-line treatment options and necessitate the use of second-line agents. The chosen regimen in this case, bedaquiline, linezolid, levofloxacin, clofazimine, and cycloserine, aligns with WHO recommendations for multidrug-resistant TB, offering high efficacy while minimizing overlapping toxicities [10]. Linezolid's excellent bone penetration and bedaquiline's potent sterilizing activity are especially advantageous in skeletal disease [11,12]. Close monitoring for adverse effects, such as myelosuppression, peripheral neuropathy, gastrointestinal disturbances, QT prolongation, and hepatotoxicity, is essential given the prolonged treatment duration; however, our patient tolerated the therapy well during the initial follow-up period.

From a public health perspective, this case reinforces several key lessons. First, in TB-endemic regions, spinal TB should remain high on the differential diagnosis for destructive vertebral lesions, even when imaging findings suggest malignancy. Second, histopathological confirmation should be obtained whenever feasible before starting potentially toxic empiric treatments such as chemotherapy or radiotherapy. Third, multidisciplinary collaboration between pulmonologists, infectious disease specialists, radiologists, and orthopedic surgeons is essential to ensure accurate diagnosis and optimized patient outcomes.

## Conclusions

This case highlights the diagnostic challenges of spinal TB, particularly when it mimics metastatic malignancy. Early consideration of TB in the differential diagnosis of destructive vertebral lesions, histopathological and microbiological confirmation via biopsy, and timely initiation of tailored multidrug-resistant TB therapy are essential. Multidisciplinary collaboration and careful imaging interpretation help prevent misdiagnosis, avoid inappropriate treatments, and optimize patient outcomes.
